# Depression is a main determinant of health-related quality of life in patients with diabetic kidney disease

**DOI:** 10.1038/s41598-022-15906-z

**Published:** 2022-07-16

**Authors:** Suhyun Kim, Junseok Jeon, Yu-Ji Lee, Hye Ryoun Jang, Eun Yeon Joo, Wooseong Huh, Yoon-Goo Kim, Dae Joong Kim, Jung Eun Lee

**Affiliations:** 1 Division of Nephrology, Department of Medicine, Hanil General Hospital, Seoul, Republic of Korea; 2 Division of Nephrology, Department of Medicine, Samsung Medical Center, Sungkyunkwan University School of Medicine, 81 Irwon-ro, Gangnam-gu, 06351 Seoul, Republic of Korea; 3 Division of Nephrology, Department of Medicine, Samsung Changwon Hospital, Sungkyunkwan University School of Medicine, 158 Paryong-ro, Masanhoewon-gu, 51353 Changwon, Republic of Korea; 4 Department of Neurology, Samsung Medical Center, Sungkyunkwan University School of Medicine, Seoul, Republic of Korea

**Keywords:** Psychology, Endocrinology, Health care, Nephrology

## Abstract

Low health-related quality of life (HRQOL) is associated with adverse outcomes in diabetic kidney disease (DKD) patients. We examined the modifiable factors associated with low HRQOL in these patients. We enrolled 141 DKD patients. HRQOL was assessed with the Short Form 36 (SF-36) questionnaire. Low HRQOL was defined as a score > one standard deviation below the mean. Depression and anxiety were assessed with the Hospital Anxiety and Depression Scale (HADS-D and HDAS-A, respectively). The patients’ median age was 65 years, and 73% were men. The prevalence rates of anxiety and depression were 8% (*n* = 11) and 17% (*n* = 24), respectively. Forty (28%) patients were identified as poor sleepers, and 40 (28%) had low physical activity levels. Anxiety, depression, and poor sleep quality were negatively correlated with SF-36 scores. Higher levels of physical activity and the estimated glomerular filtration rate (eGFR) were correlated with higher SF-36 scores, which indicated better health status. Higher depression scores (HADS-D scores) were associated with low HRQOL, independent of factors including age, sex, smoking status, comorbidities, eGFR, anemia, sleep quality, anxiety levels, and physical activity levels (odds ratio, 1.43; 95% confidence interval, 1.17–1.75). Among the clinical and psycho-physical factors, depression was a main determinant of low HRQOL in DKD patients.

## Introduction

Diabetic kidney disease (DKD) develops in approximately 40% of patients with diabetes. Despite recent therapeutic advances, the annual incidence of DKD has more than doubled over the last decade^1^. This substantial increase in prevalence is progressive as the population is aging, obesity is more prevalent, and more people survive cardiovascular problems^2^. DKD is associated with an increased risk of all-cause death and cardiovascular disease and imparts a substantial burden through health care costs as the leading cause of end-stage kidney disease (ESKD) worldwide^3^.

Health-related quality of life (HRQOL) refers to an individual’s beliefs, perceptions, experiences, and expectations for enjoyment in terms of life that may be affected by one’s health condition^4^. Patients with diabetes and kidney disease have lower HRQOL than the general population. Additionally, it is now widely accepted that low HRQOL is associated with an increased risk of cardiovascular diseases and death in patients with chronic kidney disease (CKD)^5,6^. A better understanding of HRQOL would allow clinicians to provide patient-centered care and improve long-term outcomes. However, although low HRQOL is increasingly recognized as an important predictor of adverse outcomes, previous studies have focused on nonmodifiable clinical factors such as age, sex, and comorbidities in association with HRQOL. Therefore, it is important to identify potentially modifiable risk factors associated with low HRQOL in patients with CKD.

This study was conducted to examine clinical factors associated with HRQOL using the Short Form 36 (SF-36) Health Survey Questionnaire in patients with DKD. In particular, we focused on modifiable factors such as depression, anxiety, sleep quality, anemia, glycemic control, and physical activity.

## Methods

### Study design and study subjects

We recruited outpatients with DKD between April 2017 and March 2018 from the nephrology clinic of a single tertiary hospital. The inclusion criteria were predialysis or dialysis patients with DKD who were aged ≥ 18 years and using glucose-lowering agents such as oral hypoglycemic agents or insulin. DKD was defined as biopsy-proven diabetic nephropathy or was clinically determined in diabetic patients with an estimated glomerular filtration rate (eGFR) below 60 mL/min/1.73 m^2^, with or without persistent proteinuria for 3 months or longer and without any other specific reason^7^. Persistent proteinuria was defined as a urinary albumin-to-creatinine ratio (uACR) ≥ 30 mg/g or a urinary protein-to-creatinine ratio (uPCR) ≥ 0.2 g/g. The eGFR was obtained using the Chronic Kidney Disease Epidemiology Collaboration creatinine equation^8^. All clinical studies were conducted in accordance with the guidelines of the 2013 Declaration of Helsinki. All patients signed informed consent forms. The study protocol was approved by the Institutional Review Board of the Samsung Medical Center.

### Data collection and measures

Demographic data on age, sex, body mass index (BMI), smoking status, comorbidities, medication history, diabetes duration, and dialysis were collected through an interview and medical chart review at baseline enrollment. Comorbidity burden was measured using the modified Charlson Comorbidity Index (CCI), which included the following comorbid conditions: myocardial infarction, congestive heart failure, peripheral vascular disease, cerebral vascular accident, dementia, chronic obstructive pulmonary disease, connective tissue disease, peptic ulcer disease, liver disease, diabetes mellitus, renal insufficiency, leukemia, lymphoma, solid tumor, liver disease, and AIDS/HIV^9^. The age factor was excluded from the index to examine the influence of age on HRQOL independent of comorbidities. Anthropometric and blood pressure measurements were performed by trained personnel, and laboratory data, including white blood cell counts, hemoglobin (Hb) levels, serum creatinine levels, HbA1c levels, and uACR or uPCR values, were measured at baseline. For dialysis patients, the above measurements were performed midweek before an HD (hemodialysis) session.

Health-related questionnaires were administered with the aid of study personnel. For enrolled dialysis patients, the questionnaires were answered midweek before an HD session. Sleep quality was measured using the Korean version of the Pittsburgh Sleep Quality Index (PSQI-K), which is comparable to the original version. It is a valid and reliable screening tool to identify “good” and “poor” sleepers^10^. The PSQI consists of 18 questions covering sleep quality, sleep-onset latency, sleep efficiency, sleep duration, sleep disturbance, sleeping medication use, and daytime dysfunction. Each item is scored from 0 to 3, yielding a global PSQI score ranging from 0 to 21, with higher scores indicating lower sleep quality. A total score > 5 indicates poor sleep, while a total score ≤ 5 indicates good sleep. To determine anxiety and depression levels, the Hospital Anxiety and Depression Scale (HADS) was used^11,12^. This scale is divided into an anxiety subscale (HADS-A) and a depression subscale (HADS-D), both containing seven intermingled items. Each item has 4 response categories ranging from 0 to 3, with total scores ranging from 0 to 21. For both anxiety and depression, scores of 0–7 are considered normal, 8–10 are considered borderline abnormal, and 11–21 are considered abnormal^12^. The Korean version of the International Physical Activity Questionnaire (IPAQ)-Short Form records physical activity in the last seven days^13^. The 7-item IPAQ was used to identify the total minutes spent on moderate- and vigorous-intensity physical activity, walking, and inactivity over the last seven days. Responses were converted to metabolic equivalent task minutes per week (MET-min/wk) measures according to the IPAQ scoring protocol^14^. Physical activity levels were categorized as low, moderate, and high. Low physical activity was defined as inactivity or some activity that was not enough to fall into categories 2 or 3. Moderate physical activity was defined as any of the following three criteria: three or more days of vigorous activity with at least 20 min per day; five or more days of moderate-intensity activity or walking with at least 30 min per day; or five or more days of any combination of walking, moderate-intensity, or vigorous-intensity activities achieving a minimum of at least 600 MET-min/wk. High physical activity was defined as any one of the following criteria: vigorous-intensity activity for at least three days and accumulating at least 1500 MET-min/wk or seven or more days of any combination of walking, moderate- or vigorous-intensity activities and accumulating at least 3000 MET-min/wk.

### HRQOL assessment

The SF-36 (version 2.0) was used to assess comprehensive HRQOL^15,16^. It comprises 36 questions, eight scales (physical functioning [PF], role limitation caused by physical problems [RF], bodily pain [BP], general health [GH], vitality [VT], social functioning [SF], role limitation caused by emotional problems [RE], and mental health [MH]), and two summary measures. Responses to each question were transformed into SF-36 equivalent scores, and scores ranged from 0 to 100, with higher numerical scores indicating better HRQOL. The PF, RF, BP, and GH subscales were summarized in a physical component summary (PCS), while the VT, SF, RE and MH subscales were summarized in a mental component summary (MCS). We defined low HRQOL as an SF-36 score > one standard deviation (SD) below the mean, referring to previous studies with CKD patients^17,18^. Likewise, we defined low physical health and low mental health as PCS scores and MCS scores > one SD below the mean, respectively.

### Statistical analyses

Data are expressed as percentiles for categorical variables, and medians and interquartile ranges (IQRs) for continuous variables. Chi-squared analysis or Fisher's exact test for categorical variables and the Kruskal–Wallis test for continuous variables were used to determine differences in baseline characteristics according to CKD stages. Differences in SF-36, PCS, and MCS scores between good sleepers and poor sleepers were assessed using the Mann–Whitney rank sum test. The Jonckheere–Terpstra test was used to determine trend significance in continuous variables. Spearman’s correlation coefficients were used to examine relationships between clinical parameters and SF-36 scores. The association between low HRQOL assessed by the SF-36 score and clinical factors was investigated using multivariable logistic regression analysis adjusted for age, sex, CCI score, smoking status, Hb level, eGFR and PSQI-K, HADS-A, HADS-D, and IPAQ scores, and the results were reported as odds ratios (ORs) and 95% confidence intervals (95% CIs). For the multiple logistic regression analysis, we selected confounding factors based on potential confounding factors that were proven in previous studies, the variables that showed significant association with low HRQOL in our univariate analysis, and our variables of interest. The goodness of fit of the logistic regression models was assessed using the area under the ROC curve (AUC)^19^. The multicollinearity of the variables was assessed using the variance inflation factor (VIF) with a reference value of 10 before interpreting the final output. Likewise, the association of clinical factors with low HRQOL, as assessed by the PCS score and MCS score, was investigated using the aforementioned methods. Additional logistic regression analyses were performed excluding dialysis patients since dialysis treatment itself could be a mediator in the association between HRQOL and clinical factors. Because of the lack of a solid definition of low HRQOL, additional sensitivity analyses with the aforementioned adjustments were performed using a linear regression model for the association of clinical factors with the SF-36 score as a continuous variable. Cook’s distance^20^ was used in regression analysis to find influential outliers in a set of predictor variables. There were no missing data or significant outliers. All statistical analyses were performed using SPSS version 25.0 for Windows (IBM, Armonk, NY, USA), SAS version 9.4 (SAS Institute, Cary, NC) and R 4.0.3 (Vienna, Austria; http://www.R-project.org/). A *p* value < 0.05 was considered significant.

## Results

### Study participants’ characteristics

A total of 141 patients with DKD were enrolled and completed the SF-36 and other questionnaires (Supplementary Fig. 1). The median age was 65 (57–72) years, and 103 (73%) patients were male. Fifty-six patients were categorized as having CKD stage 3, 41 were categorized as having stage 4, and 44 were categorized as having stage 5, including 28 patients on hemodialysis. Overall, the prevalence rates of anxiety and depression were 8% (*n* = 11) and 17% (*n* = 24), respectively, based on a HADS score of ≥ 8. Among a total of 24 depressive patients, 7 (29%) were on dialysis. Forty (28%) patients were identified as poor sleepers, and 40 (28%) had low physical activity levels.

The baseline characteristics of the patients according to CKD stage are summarized in Table 1. The CCI scores were higher in patients with advanced CKD stages (*p* < 0.001 for trend). Similarly, the PSQI-K scores were higher in patients with advanced CKD stages (stage 3, 2.0 [1.0–5.0]; stage 4, 2.0 [1.0–5.5]; stage 5, 4.0 [2.3–8.0], *p* = 0.005 for trend). Twenty-three percent (*n* = 13) of the patients with CKD stage 3, 24% (*n* = 10) of those with stage 4, and 39% (*n* = 17) of those with stage 5 were poor sleepers (*p* = 0.1 for trend). Anxiety scores, depression scores, and physical activity levels did not differ by CKD stage. SF-36 scores decreased progressively with advanced CKD stages (stage 3, 78.8 [71.0–82.1]; stage 4, 70.5 [56.4–81.5]; stage 5, 69.8 [56.9–81.6], *p* = 0.012 for trend).

**Table 1:** Baseline characteristics of patients with diabetic kidney disease.

Variable	Stages of chronic kidney disease			*P*
	3 (*N* = 56)	4 (*N* = 41)	5 (*N* = 44)	
Age, years	67 (58–74)	66 (59–71)	65 (55–69)	0.334
Male, n (%)	42 (75)	24 (59)	37 (84)	0.027
**Smoking, pack/years**	0.7 (0.0–3.0)	0.0 (0.0–2.0)	2.1 (0.0–4.4)	0.067
Nonsmoker, n (%)	25 (45)	21 (51)	15 (34)	0.465
Ex-smoker	24 (43)	13 (32)	20 (46)	
Current smoker	7 (12)	7 (17)	9 (20)	
**Alcohol use, n (%)**				
≤ 1/month	45 (80)	36 (88)	37 (84)	0.720
1/week	4 (7)	1 (2)	1 (2)	
2–3/week	7 (13)	3 (7)	5 (12)	
≥ 4/week	0 (0)	1 (3)	1 (2)	
**BMI, kg/m**^**2**^	25.3 (22.5–27.1)	25.0 (21.9–28.7)	25.5 (23.0–27.8)	0.827
Normal, n (%)	15 (27)	13 (32)	11 (25)	0.963
Overweight, n (%)	10 (18)	7 (17)	9 (20)	
Obesity, n (%)	31 (55)	21 (51)	24 (55)	
SBP, mmHg	130 (120–140)	132 (127–142)	137 (124–148)	0.303
DBP, mmHg	76 (67–82)	76 (65–84)	72 (65–77)	0.281
DM duration, years	19.0 (13.3–27.0)	19.0 (14.0–27.5)	17.5 (11.3–22.0)	0.393
**Comorbidities, n (%)**				
MI	4 (7)	5 (12)	2 (5)	0.410
CHF	2 (4)	4 (10)	6 (14)	0.179
PAOD	5 (9)	3 (7)	3 (7)	0.918
Stroke	11 (20)	9 (22)	6 (14)	0.870
Dementia	0 (0)	2 (5)	0 (0)	0.083
COPD	2 (4)	0 (0)	1 (2)	0.779
Malignancy	6 (11)	5 (12)	6 (14)	0.905
Chronic liver disease	13 (23)	9 (22)	8 (18)	0.824
Peptic ulcer disease	0 (0)	3 (7)	5 (11)	0.028
**CCI score**	6.0 (5.0–7.0)	6.0 (5.0–7.0)	7.0 (6.0–8.0)	< 0.001
Low, n (%)	5 (9)	1 (2)	0 (0)	< 0.001
Moderate, n (%)	35 (62)	22 (54)	12 (27)	
High, n (%)	10 (18)	12 (29)	14 (32)	
Very high, n (%)	6 (11)	6 (14)	18 (41)	
**PSQI-K score**	2.0 (1.0–5.0)	2.0 (1.0–5.5)	4.0 (2.3–8.0)	0.002
Good sleeper, n (%)	43 (77)	31 (76)	27 (61)	0.189
Poor sleeper, n (%)	13 (23)	10 (24)	17 (39)	
**Anxiety score**	2.0 (1.0–3.0)	3.0 (1.0–4.0)	2.0 (1.0–6.0)	0.427
Normal, n (%)	53 (95)	38 (93)	39 (89)	0.695
Borderline, n (%)	3 (5)	2 (5)	4 (9)	
Abnormal, n (%)	0 (0)	1 (2)	1 (2)	
**Depression score**	3.0 (2.0–5.0)	4.0 (2.5–6.0)	4.0 (1.0–8.0)	0.259
Normal, n (%)	50 (89)	35 (85.5)	32 (73)	0.252
Borderline, n (%)	4 (7)	5 (12)	8 (18)	
Abnormal, n (%)	2 (4)	1 (2.5)	4 (9)	
**IPAQ score**	1,680 (810–3,360)	1,560 (120–3,360)	840 (480–2200)	0.263
Low, n (%)	12 (21)	15 (37)	13 (29)	0.122
Moderate, n (%)	28 (50)	14 (34)	25 (57)	
High, n (%)	16 (29)	12 (29)	6 (14)	
**SF-36 score**	78.8 (71.0–82.1)	70.5(56.4–81.5)	69.8(56.9–81.6)	0.029
Physical functioning	90.0 (85.0–95.0)	85.0 (62.5–95.0)	85.0 (61.3–90.0)	0.047
Role-physical	75.0 (56.3–100.0)	50.0 (25.0–100.0)	50.0 (25.0–100.0)	0.002
Bodily pain	100.0 (90.0–100.0)	100.0 (83.8–100.0)	100.0 (81.9–100.0)	0.277
Social functioning	100.0 (90.6–100.0)	100.0 (75.0–100.0)	100.0 (87.5–100.0)	0.260
Mental health	72.0 (64.0–80.0)	76.0 (64.0–80.0)	72.0 (56.0–76.0)	0.155
Role-emotional	100.0 (66.7–100.0)	100.0 (33.3–100.0)	100.0 (41.7–100.0)	0.244
Vitality	50.0 (45.0–60.0)	50.0 (45.0–62.5)	50.0 (40.0–60.0)	0.295
General health	40.0 (21.3–65.0)	40.0 (25.0–57.5)	40.0 (21.3–60.0)	0.991
PCS score	78.8 (65.2–85.0)	71.3 (52.8–82.5)	65.0 (51.9–79.4)	0.011
MCS score	80.5 (67.5–83.9)	74.4 (59.1–82.5)	74.3 (51.7–82.9)	0.208
Hb, g/dL	12.7 (11.6–14.4)	11.9 (10.6–12.7)	11.1 (10.3–12.1)	< 0.001
eGFR, mL/min/1.73 m^2^	41 (37–47)	22 (18–27)	7 (6–11)	< 0.001
HbA1c, %	6.9 (6.2–7.7)	6.8 (6.4–7.8)	6.5 (5.8–6.9)	0.014
uPCR, mg/mg Cr	0.97 (0.53–2.13)	2.40 (1.08–4.44)	3.78 (1.10–5.45)	0.001

Values are expressed as the median (interquartile range) or percentage, as appropriate.

The eight SF-36 scales are as follows: physical functioning (PF), role limitation caused by physical problems (RF), bodily pain (BP), general health (GH), vitality (VT), social functioning (SF), role limitation caused by emotional problems (RE), and mental health (MH).

*BMI* body mass index, *CCI* Charlson Comorbidity Index, *CHF* congestive heart failure, *COPD* chronic obstructive lung disease, *DBP* diastolic blood pressure, *DM* diabetes mellitus, *eGFR* estimated glomerular filtration rate, *IPAQ* International Physical Activity Questionnaire, *Hb* hemoglobin, *MCS* mental component summary, *MI* myocardial infarction, *PAOD* peripheral artery occlusive disease, *PCS* physical component summary, *PSQI-K* Korean version of the Pittsburgh Sleep Quality Index, *SBP* systolic blood pressure, *SF-36* Short Form 36 Health Survey Questionnaire, *uPCR* urine protein-to-creatinine ratio.

### SF-36 score distributions

**Figure. 1:**
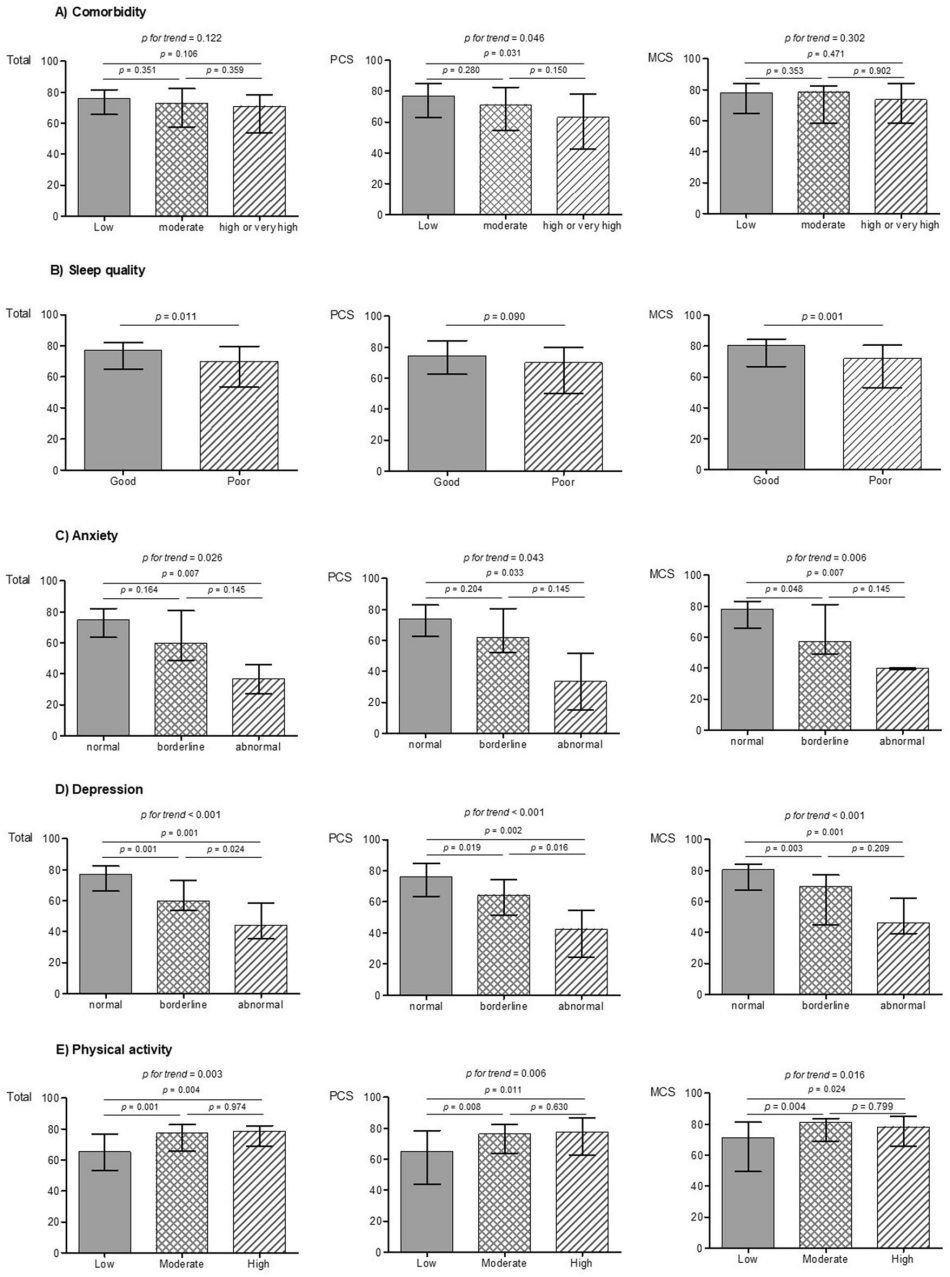
Distribution of health-related quality of life according to comorbidities (**A**), sleep quality (**B**), anxiety (**C**), depression (**D**), and physical activity level (**E**) among patients with diabetic kidney disease. Bars and error bars indicate the median value and interquartile range, respectively. Abbreviations: *CCI* Charlson Comorbidity Index, *MCS* mental component summary, *SF-36* Short Form 36 of life Health Survey Question, *SQ* sleep quality, *PA* physical activity, *PCS* physical component summary.

Distributions of SF-36 scores according to CCI scores, sleep quality, anxiety, depression, and physical activity levels are presented in Fig. 1. SF-36 scores did not differ according to CCI scores (*p* = 0.122; Fig. 1a). Conversely, poor sleepers showed lower HRQOL scores than good sleepers; the median (IQR) SF-36 total score was 69.9 (53.3–79.6) and 77.0 (64.8–82.2; *p* = 0.011) for poor and good sleepers, respectively (Fig. 1b). The SF-36 total score was progressively lower in patients with higher HADS-A scores; the SF-36 total scores were 74.8 (63.6–82.0), 59.8 (48.7–80.1), and 36.7 (27.2–46.1) in patients with normal, borderline abnormal, and abnormal HADS-A scores, respectively (*p* = 0.026 for trend) (Fig. 1c). Similarly, patients with higher HADS-D scores showed progressively lower SF-36 total scores; the SF-36 total scores were 77.0 (66.1–82.3), 59.8 (53.5–73.1), and 44.1 (35.2–58.4) in patients with normal, borderline abnormal, and abnormal HADS-D scores, respectively (*p* < 0.001 for trend) (Fig. 1d). The subjects with higher physical activity levels showed progressively higher SF-36 total scores; the SF-36 total scores were 65.4 (53.3–76.9), 77.6 (65.8–82.8), and 78.6 (68.9–82.1] in patients with low, moderate, and high physical activity levels, respectively (*p* = 0.003 for trend) (Fig. 1e).

### Association between clinical factors and HRQOL

Correlations between clinical variables and HRQOL scores were analyzed. As depicted in Table 2, SF-36 total scores were negatively correlated with anxiety, depression, and sleep quality. HRQOL scores were positively correlated with the eGFR and physical activity levels.

**Table 2:** Correlation between HRQOL and clinical characteristics.

Parameter	SF-36 score		PCS score		MCS score	
	Correlation coefficient (*r*)	*P*	Correlation coefficient (*r*)	*P*	Correlation coefficient (*r*)	*P*
Age, years	0.118	0.163	0.026	0.759	0.221	0.008
Sex, female	− 0.158	0.062	− 0.139	0.100	− 0.106	0.212
BMI, kg/m^2^	− 0.077	0.365	− 0.104	0.218	− 0.061	0.475
SBP, mmHg	0.123	0.145	0.052	0.543	0.116	0.170
DM duration, years	0.029	0.729	0.029	0.733	0.010	0.911
Smoking status	0.092	0.279	0.131	0.121	− 0.002	0.985
CCI score	− 0.092	0.279	− 0.136	0.109	− 0.034	0.691
Hb, g/dL	0.097	0.251	0.104	0.220	0.041	0.632
eGFR, mL/min/1.73 m^2^	0.189	0.025	0.223	0.008	0.111	0.190
HbA1c, %	− 0.043	0.616	− 0.056	0.511	− 0.044	0.605
PSQI-K score	− 0.200	0.017	− 0.153	0.071	− 0.218	0.009
Sleep duration, hours	0.103	0.225	0.038	0.653	0.145	0.086
HADS-A score	− 0.276	0.001	− 0.142	0.092	− 0.406	< 0.001
HADS-D score	− 0.540	< 0.001	− 0.446	< 0.001	− 0.508	< 0.001
IPAQ score	0.285	0.001	0.257	0.002	0.234	0.005

*BMI* body mass index, *CCI* Charlson Comorbidity Index, *DM* diabetes mellitus, *eGFR* estimated glomerular filtration rate, *IPAQ* International Physical Activity Questionnaire, *HADS-A* Hospital Anxiety and Depression Scale-anxiety subscale, *HADS-D* Hospital Anxiety and Depression Scale-depression subscale, *Hb* hemoglobin, *HRQOL* health-related quality of life, *MCS* mental component summary, *PCS* physical component summary, *PSQI-K* Korean version of the Pittsburgh Sleep Quality Index, *SBP* systolic blood pressure, *SF-36* Short Form 36 Health Survey Questionnaire.

In the multivariable logistic regression analyses, adjusted for age, sex, smoking status, CCI score, Hb level, eGFR and PSQI-K, HADS-A, HADS-D, and IPAQ scores, that were performed to determine the factors associated with low HRQOL as assessed by the SF-36 total score, depression scores (HADS-D) were associated with low HRQOL, independent of age, sex, smoking status, comorbidities (CCI score), eGFR, anemia, sleep quality (PSQI-K score), anxiety (HADS-A score), and physical activity level (IPAQ score); the OR (95% CI) per 1-score increment was 1.43 (1.17–1.75) (*p* < 0.001) (Table 3). However, the association of the eGFR, PSQI-K score, anxiety score, and IPAQ score with low HRQOL was attenuated in the multivariable logistic regression analysis. In particular, the HADS-D score showed an evident association with physical health; the OR (95% CI) per 1-score increment of HADS-D scores was 1.36 (1.13–1.64) (*p* = 0.001). However, the association between the HADS-D score and mental health was attenuated. Rather, anxiety scores (HADS-A) were independently associated with low mental health (Table 3). Our fitting model showed no significant difference between the model and the observed data (AUC value, 0.8515 for overall HRQOL; 0.7949 for Physical health; 0.8511 for Metal health). The VIF showed no evidence of a multicollinearity problem among the independent variables (all VIFs < 3).

**Table 3:** Determinants of low health-related quality of life (HRQOL) among patients with diabetic kidney disease by multivariable logistic regression analyses.

HRQOL	Variable	Odds ratio	95% CI	*P*
Overall HRQOL	Age, years	0.98	0.91–1.05	0.544
	Sex, female	4.05	0.98–16.8	0.053
	Smoking status	0.41	0.09–1.75	0.228
	CCI score	1.18	0.77–1.80	0.449
	Hb, g/dL	0.92	0.64–1.33	0.665
	eGFR, mL/min/1.73 m^2^	0.98	0.93–1.03	0.413
	PSQI-K score	1.09	0.92–1.28	0.317
	HADS-A score	1.08	0.88–1.31	0.482
	HADS-D score	1.43	1.17–1.75	< 0.001
	IPAQ score	1.00	1.00–1.00	0.922
Physical health	Age, years	1.01	0.95–1.08	0.734
	Sex, female	2.17	0.56–8.51	0.265
	Smoking status	0.62	0.16–2.36	0.484
	CCI score	1.14	0.77–1.67	0.520
	Hb, g/dL	1.10	0.77–1.56	0.612
	eGFR, mL/min/1.73 m^2^	0.99	0.95–1.03	0.565
	PSQI-K score	1.12	0.96–1.31	0.146
	HADS-A score	0.95	0.78–1.15	0.588
	HADS-D score	1.36	1.13–1.64	0.001
	IPAQ score	1.00	1.00–1.00	0.972
Mental health	Age, years	0.94	0.87–1.02	0.127
	Sex, female	2.53	0.58–11.09	0.220
	Smoking status	0.82	0.20–3.33	0.780
	CCI score	1.27	0.79–2.03	0.327
	Hb, g/dL	1.28	0.86–1.90	0.218
	eGFR, mL/min/1.73 m^2^	0.96	0.91–1.01	0.141
	PSQI-K score	1.13	0.94–1.34	0.197
	HADS-A score	1.34	1.10–1.64	0.004
	HADS-D score	1.17	0.97–1.41	0.111
	IPAQ score	1.00	1.00–1.00	0.694

Low HRQOL was defined as an SF-36 score > one SD below the mean. Low physical health and low mental health were defined as PCS scores and MCS scores > one SD below the mean, respectively.

*CCI* Charlson Comorbidity Index, *eGFR* estimated glomerular filtration rate, *IPAQ* International Physical Activity Questionnaire, *HADS-A* Hospital Anxiety and Depression Scale-anxiety subscale, *HADS-D* Hospital Anxiety and Depression scale-depression subscale, *Hb* hemoglobin, *HRQOL* health-related quality of life, *MCS* mental component summary, *PCS* physical component summary, *PSQI-K* Korean version of the Pittsburgh Sleep Quality Index, *SF-36* Short Form 36 Health Survey Questionnaire.

### Sensitivity analyses

We performed sensitivity analyses to confirm the robustness of the results. In linear regression analysis, the HADS-D score was the most potent modifiable factor associated with HRQOL; a higher HADS-D score was associated with a lower SF-36 total score, PCS score, and MCS score (Supplementary Table 1).

In the analyses that excluded dialysis patients (*n* = 28), a consistent association between depression and low HRQOL was observed; the ORs (95% CIs) per 1-score increment of HADS-D scores were 1.43 (1.14–1.80) and 1.31 (1.07–1.60) for low HRQOL as assessed by the SF-36 total score and PCS score, respectively (all *p* < 0.05, Supplementary Table 2). Additionally, a consistent association between anxiety and low mental health was observed; the OR (95% CI) per 1-score increment of HADS-A scores was 1.32 (1.03–1.69) (*p* = 0.031).

## Discussion

We examined clinical and psycho-physical factors, including health-related behaviors associated with HRQOL, in patients with DKD stage 3–5, focusing especially on modifiable factors. We found that depression was a main determinant of low HRQOL in these patients, independent of factors including age, sex, comorbidities, smoking status, kidney function, anemia, glycemic control level, sleep quality, anxiety, and physical activity level. This association between depression and low HRQOL, in particular, was evident in the physical health domain.

Approximately 20–40% of patients with predialysis CKD, as well as those on maintenance dialysis, have depression^21^. Diabetes in particular is known to be a risk factor for depression in patients with CKD^22^. Moreover, DKD progression is associated with an increased risk of depression^23^. In patients with CKD or ESKD, depression is associated with increased health-care utilization, lower treatment compliance, and poor social and occupational role functioning^24–26^. Moreover, although HRQOL is an important patient-centered outcome, and low HRQOL is increasingly recognized as an important predictor of adverse outcomes such as progression to ESKD and all-cause and cardiovascular mortalities in patients with CKD, previous studies have largely focused on nonmodifiable clinical factors such as age, sex, and comorbidities in association with HRQOL. Depression as a modifiable factor has been shown to have a negative effect on HRQOL in patients with diabetes^27^ and CKD^28,29^. Some authors even reported that depressive symptoms were more associated with low HRQOL than measures of dialysis adequacy, other demographic variables, and low hemoglobin levels^30,31^. However, the association between depression and low HRQOL shown in previous studies might have been affected by health-related behaviors such as sleep quality, physical activity, and smoking status. Low physical activity levels and poor sleep quality are known to be associated with low HRQOL in patients with CKD^32,33^. In this study, low physical activity levels and poor sleep quality were also associated with low HRQOL. However, after adjusting for nonmodifiable and modifiable factors, depression was the most relevant modifiable factor associated with low HRQOL. In particular, we found that depression was more obviously associated with low physical health than with low mental health. Similar to our results, a previous study^34^ showed that the PCS score was more impaired than the MCS score in patients with predialysis CKD, and the PCS score was negatively associated with depression in multivariate analysis. When depression is added to the diabetic state, the effect of depression on HRQOL can be more additive for the PCS score than the MCS score^35^. In addition to depression, there are several sociodemographic and clinical factors associated with low HRQOL in patients with CKD. The Chronic Renal Insufficiency Cohort (CRIC) and Hispanic CRIC studies have reported that younger age, female sex, diabetes, peripheral vascular disease, congestive heart failure, low education level, obesity, and lower eGFR were significantly associated with low HRQOL in large diverse CKD cohorts^5^. Anemia may also negatively affect HRQOL, and hemoglobin correction is associated with improved HRQOL in patients with CKD^36^. In the present study, however, only depression was associated with low HRQOL in multivariate analysis.

This study has some limitations that should be considered in the interpretation of the results. First, our findings are limited in the assessment of causal relationships between depression and HRQOL because the study was cross-sectional. Therefore, the possibility of reverse causality between low HRQOL and depression may exist. However, most previous studies of patients with CKD have demonstrated the role of depression as a risk factor for low HRQOL. Moreover, low HRQOL as a risk factor for depression is rather likely to act as a mediator in the association between various risk factors and depression. In addition, although we adjusted for confounding factors in the analyses for the association with the quality of life in patients with DKD, unmeasured factors may still remain. In particular, factors such as marital status, education level, and income level may influence quality of life but these factors were not addressed in this study. Second, this study included dialysis patients. Dialysis treatment itself can reduce HRQOL and increase symptoms such as anxiety, depression, and sleep disorders, and may act as a confounding factor in the association between these factors and HRQOL. However, when we performed the sensitivity analysis excluding dialysis patients, the results did not change. Third, we used the HADS instead of structured clinical interviews to assess depression and anxiety; hence, misclassification was possible. Nevertheless, the HADS is known to be a reliable and valid tool for identifying depression in patients with CKD and ESKD. Moreover, this misclassification error was nondifferential, which would likely bias our results toward the null. Last, this was a single-center study with a relatively small sample size. Moreover, DKD patients with an eGFR ≥ 60 mL/min/1.73 m^2^ were excluded from this study, therefore limiting the generalizability of our results, especially for DKD populations with preserved renal function. Therefore, our study highlights the potential need for randomized controlled trials to assess the temporal relationship between depression and low HRQOL in patients with DKD and to further evaluate the effect of early detection of depression and appropriate depression care on the health outcomes of these patients. Notwithstanding these limitations, the strength of our study is that we conducted comprehensive analyses including health-related behaviors, such as smoking, sleep quality, and physical activity, as well as modifiable and nonmodifiable clinical characteristics to examine factors associated with HRQOL in patients with DKD.

## Conclusion

This study investigated various factors associated with low HRQOL in patients with DKD and identified depression as a major determinant of reduced quality of life. These findings reinforce the importance of assessing depression in patients with DKD and incorporating this assessment into clinicians’ management decisions, urging future studies to provide biological rationale and evaluate causality.

## Supplementary Information

Supplementary Information.

## Data Availability

The datasets generated during and/or analyzed during the current study are available from the corresponding author upon reasonable request.
