# Supplementary material for: Depression is a main determinant of health-related quality of life in patients with diabetic kidney disease
Source: Sci Rep. 2022 Jul 16;12:12159. doi: 10.1038/s41598-022-15906-z (PMC9288542; doi:10.1038/s41598-022-15906-z)
Supplement: Supplementary file 1 — Supplementary Information. [file 41598_2022_15906_MOESM1_ESM.docx]

**Supplementary Materials**

**Table S1**. Factors associated with low HRQOL among patients with diabetic kidney disease by linear regression model (N = 141)

| SF-36 | Variable | Univariable Analysis | | Multivariable Analysis* | |
| --- | --- | --- | --- | --- | --- |
|  |  | Coefficient (95% CI) | *p* | Coefficient (95% CI) | *p* |
| Total | Age, years | -4.726(-10.329 – 0.878) | 0.098 |  |  |
|  | Sex, female | 0.173(-0.041 – 0.388) | 0.112 | -6.747(-11.396 – -2.099) | 0.005 |
|  | Smoking status | 2.675(-2.374 – 7.724) | 0.297 |  |  |
|  | CCI score | -0.388(-1.703 – 0.927) | 0.561 |  |  |
|  | Hb, g/dL | 0.563(-0.900 – 2.028) | 0.448 |  |  |
|  | eGFR, mL/min/1.73 m^2^ | 0.212(0.056 – 0.369) | 0.008 |  |  |
|  | PSQI-K score | -1.103(-1.809 – -0.397) | 0.002 | -0.683(-1.283 – -0.084) | 0.026 |
|  | HADS-A score | -1.948(-2.793 – -1.103) | <0.001 |  |  |
|  | HADS-D score | -2.488(-3.144 – -1.831) | <0.001 | -2.517(-3.159 – -1.875) | <0.001 |
|  | IPAQ score | 0.001(0.000 – 0.002) | 0.063 |  |  |
| PCS | Age, years | 0.016(-0.250 – 0.283) | 0.904 |  |  |
|  | Sex, female | -6.730(-13.610 – 0.150) | 0.055 | -11.083(-17.130 –-5.036) | <0.001 |
|  | Smoking status | 4.462(-1.737– 10.661) | 0.157 |  |  |
|  | CCI score | -1.319(-2.926 – 0.288) | 0.107 | -2.099(-3.496 – -0.702) | 0.004 |
|  | Hb, g/dL | 1.006(-0.792 – 2.804) | 0.271 |  |  |
|  | eGFR, mL/min/1.73 m^2^ | 0.265(0.073 – 0.458) | 0.007 |  |  |
|  | PSQI-K score | -1.049(-1.931 – -0.167) | 0.020 |  |  |
|  | HADS-A score | -1.423(-2.513 – -0.332) | 0.011 |  |  |
|  | HADS-D score | -2.543(-3.401 – -1.685) | <0.001 | -2.852(-3.676 – -2.029) | <0.001 |
|  | IPAQ score | 0.001(0.000 – 0.002) | 0.099 |  |  |
| MCS | Age, years | 0.331(0.109 – 0.552) | 0.004 |  |  |
|  | Sex, female | -2.742(-8.697 – 3.214) | 0.364 |  |  |
|  | Smoking status | 0.888(-4.459 – 6.235) | 0.743 |  |  |
|  | CCI score | 0.546(-0.840 – 1.933) | 0.437 |  |  |
|  | Hb, g/dL | 0.125(-1.423 – 1.672) | 0.874 |  |  |
|  | eGFR, mL/min/1.73 m^2^ | 0.159(-0.008 – 0.326) | 0.062 |  |  |
|  | PSQI-K score | -1.160(-1.906 – -0.414) | 0.003 | -0.853(0.009 – -1.490) | 0.009 |
|  | HADS-A score | -2.469(-3.330 – -1.607) | <0.001 | -1.538(-2.402 – -0.674) | <0.001 |
|  | HADS-D score | -2.433(-3.145 – -1.720) | <0.001 | -1.745(-2.492 – -0.997) | <0.001 |
|  | IPAQ score | 0.001(0.000 – 0.002) | 0.110 |  |  |

*Stepwise methods, R^2^: Overall HRQOL, 0.359; Physical health, 0.295; Mental health, 0.341

CCI, Charlson Comorbidity Index; CI, Confidence interval; eGFR, estimated glomerular filtration rate; IPAQ, International Physical Activity Questionnaire; HADS-A, Hospital Anxiety and Depression Scale-anxiety subscale; HADS-D, Hospital Anxiety and Depression scale-depression subscale; Hb, hemoglobin; HRQOL, health-related quality of life; PSQI-K, Korean version of the Pittsburgh Sleep Quality Index; SF-36, Short Form 36 Health Survey Questionnaire.

**Table S2.** Multivariable logistic regression analyses of low HRQOL in non-dialysis DKD patients (*n* = 113)

| HRQOL | Variable | Odds ratio | 95% CI | *P* |
| --- | --- | --- | --- | --- |
| Overall HRQOL | Age, years | 0.97 | 0.88–1.06 | 0.509 |
|  | Sex, female | 5.22 | 1.01–24.88 | 0.038 |
|  | Smoking status | 0.62 | 0.12–3.13 | 0.560 |
|  | CCI score | 1.16 | 0.61–2.19 | 0.655 |
|  | Hb, g/dL | 0.77 | 0.49–1.22 | 0.263 |
|  | eGFR, mL/min/1.73 m^2^ | 0.97 | 0.91–1.03 | 0.333 |
|  | PSQI-K score | 0.96 | 0.79–1.17 | 0.710 |
|  | HADS-A score | 0.97 | 0.76–1.24 | 0.829 |
|  | HADS-D score | 1.43 | 1.14–1.80 | 0.002 |
|  | IPAQ score | 1.00 | 1.00–1.00 | 0.370 |
| Physical health | Age, years | 1.00 | 0.92–1.08 | 0.989 |
|  | Sex, female | 2.84 | 0.65–12.33 | 0.164 |
|  | Smoking status | 0.81 | 0.18–3.61 | 0.782 |
|  | CCI score | 1.12 | 0.65–1.91 | 0.687 |
|  | Hb, g/dL | 0.93 | 0.61–1.41 | 0.715 |
|  | eGFR, mL/min/1.73 m^2^ | 0.99 | 0.94–1.05 | 0.814 |
|  | PSQI-K score | 1.03 | 0.86–1.23 | 0.774 |
|  | HADS-A score | 0.90 | 0.72–1.13 | 0.345 |
|  | HADS-D score | 1.31 | 1.07–1.60 | 0.010 |
|  | IPAQ score | 1.00 | 1.00–1.00 | 0.599 |
| Mental health | Age, years | 0.92 | 0.83–1.01 | 0.072 |
|  | Sex, female | 3.27 | 0.62–17.39 | 0.164 |
|  | Smoking status | 0.94 | 0.18–4.90 | 0.942 |
|  | CCI score | 1.45 | 0.80–2.63 | 0.227 |
|  | Hb, g/dL | 1.18 | 0.75–1.84 | 0.478 |
|  | eGFR, mL/min/1.73 m^2^ | 0.96 | 0.89–1.03 | 0.253 |
|  | PSQI-K score | 0.98 | 0.78–1.23 | 0.852 |
|  | HADS-A score | 1.32 | 1.03–1.69 | 0.031 |
|  | HADS-D score | 1.10 | 0.88–1.37 | 0.396 |
|  | IPAQ score | 1.00 | 1.00–1.00 | 0.293 |

Low HRQOL was defined as an SF-36 score > one SD below the mean. Low physical health and low mental health were defined as PCS scores and MCS scores > one SD below the mean, respectively.

CCI, Charlson Comorbidity Index; eGFR, estimated glomerular filtration rate; IPAQ, International Physical Activity Questionnaire; HADS-A, Hospital Anxiety and Depression Scale-anxiety subscale; HADS-D, Hospital Anxiety and Depression scale-depression subscale; Hb, hemoglobin; HRQOL, health-related quality of life; PSQI-K, Korean version of the Pittsburgh Sleep Quality Index; MCS, mental component summary; PCS, physical component summary; SF-36, Short Form 36 Health Survey Questionnaire.

**Figure S1.** Flow diagram for study participants

**
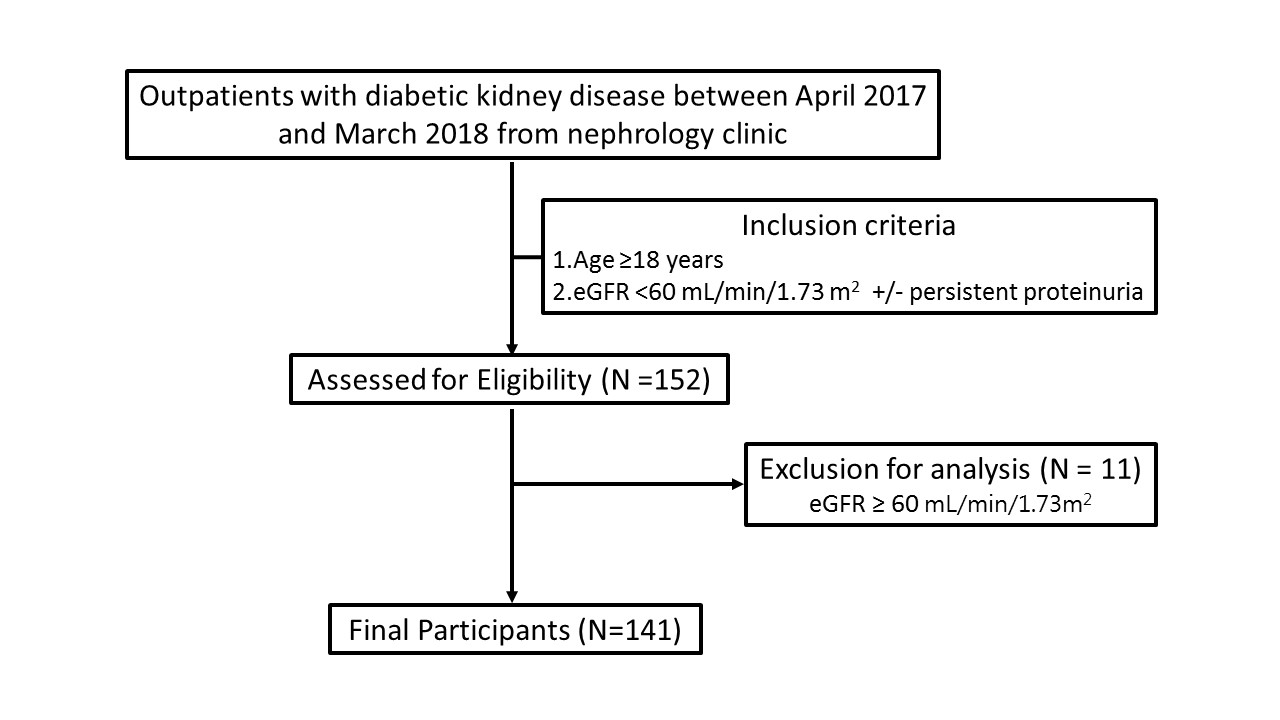
**
